# The Enigmatic Alphavirus Non-Structural Protein 3 (nsP3) Revealing Its Secrets at Last

**DOI:** 10.3390/v10030105

**Published:** 2018-02-28

**Authors:** Benjamin Götte, Lifeng Liu, Gerald M. McInerney

**Affiliations:** Department of Microbiology, Tumor and Cell Biology, Karolinska Institutet, 171 77 Stockholm, Sweden; benjamin.gotte@ki.se (B.G.); lifeng.liu@ki.se (L.L.)

**Keywords:** alphavirus, hypervariable domain, host-pathogen interaction, macro domain, polyprotein processing, replicase, replication complex

## Abstract

Alphaviruses encode 4 non-structural proteins (nsPs), most of which have well-understood functions in capping and membrane association (nsP1), polyprotein processing and RNA helicase activity (nsP2) and as RNA-dependent RNA polymerase (nsP4). The function of nsP3 has been more difficult to pin down and it has long been referred to as the more enigmatic of the nsPs. The protein comprises three domains, an N-terminal macro domain, a central zinc-binding domain and a C-terminal hypervariable domain (HVD). In this article, we review old and new literature about the functions of the three domains. Much progress in recent years has contributed to a picture of nsP3, particularly through its HVD as a hub for interactions with host cell molecules, with multiple effects on the biology of the host cell at early points in infection. These and many future discoveries will provide targets for anti-viral therapies as well as strategies for modification of vectors for vaccine and oncolytic interventions.

## 1. Introduction

Alphaviruses are a group of enveloped positive-sense single-stranded RNA (ss(+)RNA) viruses and belong to the family *Togaviridae*. They generally exhibit a broad host tropism and are transmitted to various vertebrate hosts including humans via invertebrate vectors, usually mosquitoes, in which they establish persistent and asymptomatic infections. In contrast, infection in the vertebrate host is acute and can cause severe disease, which can sometimes be persistent. Members of the genus *Alphavirus* are globally distributed and geographically and evolutionary distinguished into “Old World” and “New World” alphaviruses. Infection with New World alphaviruses, like Venezuelan equine encephalitis virus (VEEV) and Eastern equine encephalitis virus (EEEV), can result in encephalitic disease in humans. Representative members of Old World alphaviruses include the model viruses Semliki Forest virus (SFV) and Sindbis virus (SINV) as well as important human pathogens such as chikungunya virus (CHIKV) and Ross River virus (RRV). Infection with SFV causes fatal encephalitis in mice but is associated with only mild symptoms in humans. CHIKV in contrast is a re-emerging human pathogen that causes more severe symptoms like joint pain, fever, rash and arthritis [1] and case-fatality rates range from 0.1% to 11.9% during epidemics [2,3]. In infected individuals, CHIKV replicates in the skin and spreads to the liver, muscle, joints, lymphoid tissue and in some cases the brain. For several decades, chikungunya fever had been occasionally detected in tropical regions but in 2004, CHIKV emerged from the East coast of Africa causing several global outbreaks [4,5]. Since then, millions of cases in over 50 countries have been reported, including mainland USA, Italy and France. At the same time, the habitat of the *Aedes* mosquito vector is expanding, putting even larger populations under threat. In addition to CHIKV, other alphaviruses that are of public health importance are also gaining ground. Among these are Mayaro virus (MAYV), which is endemic in South America, O’nyong’nyong virus (ONNV), which causes outbreaks in Africa, RRV which is endemic in Australia, Papua New Guinea and Pacific Islands and Sindbis virus (SINV) which is linked to outbreaks in South Africa, Australia, Indonesia, Philippines, China and Northern Europe, including Sweden [6,7]. To date, no antiviral therapies or vaccines have been approved for any alphavirus, highlighting the importance to promote this area of research.

**Figure 1:**
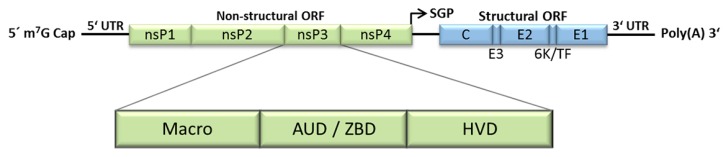
Alphavirus genome and nsP3 domains. The alphavirus genome is a positive-sense single-stranded RNA molecule, which contains two ORFs. The 5′ ORF encodes the non-structural proteins (nsP) 1–4 and the 3′ ORF encodes the structural proteins (Capsid (C), envelope glycoproteins (E1–3), a 6 kDa protein (6K) and the transframe protein (TF), which is comprised of a C-terminal extension of the 6 K protein after a frameshift event). nsP3 is subdivided into three domains, the macro domain, the alphavirus-unique or zinc-binding domain (AUD/ZBD) and the hypervariable domain (HVD). m^7^G—7-methylguanosine; UTR—untranslated region; ORF—open reading frame; SGP—subgenomic promoter; Poly(A)—polyadenylation.

Infection of cells starts with binding of the virion to its receptor, as yet unidentified for many alphaviruses. This binding is mediated by the E2 glycoprotein and induces receptor-mediated endocytosis and subsequent fusion of viral and endosomal membranes [8,9]. The nucleocapsid is released into the cytoplasm and disassembled, liberating the viral genome. The viral positive stranded RNA genome has a 5′ 7-methylguanosine cap and a 3′ poly(A) tail and contains two open reading frames (ORFs) (Figure 1). The 5′ ORF encodes the non-structural proteins nsP1–4, which together form the viral replicase. These proteins are expressed as a polyprotein, translated directly from the incoming viral genome and processed in a highly regulated manner into individual proteins by the viral protease nsP2 [10]. The structural proteins, which form the virus particle, are expressed later from a subgenomic mRNA, which is synthesized during viral replication. For more details about the alphavirus life cycle, the reader is referred to a number of extensive reviews [11,12] as well as other review articles in this special issue.

The newly-expressed non-structural polyprotein first associates with the plasma membrane (PM) where viral RNA synthesis is initiated [13,14]. In this process, viral replication complexes induce light bulb-shaped membrane evaginations, protruding outwards from the PM. The complexes, referred to as spherules are connected to the cytoplasm by a narrow bottleneck, through which ribonucleotides must flow in and nascent RNA must be exported. For some alphaviruses, like SFV, the spherules are internalized at later stages of infection and transported to the perinuclear area, where they form cytopathic vacuoles (CPV) [13]. These CPVs were shown to be composed of modified endosomes and lysosomes [15]. The viral replicase first uses the positive sense genome as template to synthesize complementary negative sense RNA which subsequently serves as template for the synthesis of genomic and subgenomic plus-strand RNA [16]. The different replicase activities are orchestrated by different processing intermediates that arise when the non-structural polyprotein is processed into the individual nsPs. The non-structural proteins fulfil different tasks during viral RNA synthesis but some are known to localize to different cellular compartments also [17,18,19]. In the replication complexes, nsP1 is required for PM-association of the replicase complex and 5′ capping of viral RNA species [20,21], nsP2 serves as RNA helicase and protease for polyprotein processing [10,22] and nsP4 is the RNA-dependent RNA polymerase [23]. NsP3 is also an essential component of the viral RNA replicase [24] and its importance for negative sense and subgenomic RNA synthesis has been reported [25,26]. However, its precise function has been enigmatic for a long time.

## 2. Expression of the Non-Structural Polyprotein Is Regulated by the Presence of Opal Stop Codon at the nsP3/4 Junction in Some Alphaviruses

NsP3 is expressed as part of a polyprotein from the 42S positive sense RNA genome [27,28,29]. For most alphaviruses, including SINV and RRV, two non-structural protein precursors are produced, the polyprotein P123 and, to a lower level, P1234. A leaky opal stop codon in the nsP3 coding sequence of these viruses restricts the expression of nsP4 and thereby limits production of the viral RNA-dependent RNA polymerase nsP4 [30,31]. Exceptions include strains of SFV and ONNV for which the termination codon is replaced by an arginine codon, thus resulting in production of P1234 exclusively [29,31].

ONNVs possess either a sense or an opal stop codon at this position and it has been suggested that both quasispecies populations might be required during the natural life cycle of ONNV [32,33]. When the Arg codon in SG650 ONNV was replaced by an opal codon, electrotransfected BHK-21 and C6/36 mosquito cells produced almost 10-fold and 500-fold less virus compared to the parental virus, respectively [33]. On the contrary, in the natural mosquito host of ONNV, *Anopheles gambiae*, the opal stop codon provides a clear fitness advantage over the Arg codon and approximately doubled the infectivity. The authors therefore concluded that by limiting the replication, ONNVs with an opal termination codon might more easily establish persistent infections in the mosquito host. Interestingly, when several ONNV isolates that possessed the opal codon were passaged in vertebrate Vero cells, all of them showed an opal-to-Arg codon change after the fifth passage [32], suggesting that different hosts might confer different selective pressure on ONNV quasispecies.

For CHIKV, the majority of strains contain an opal termination codon [34,35] but isolates that encode an Arg codon in that position have also been found [36,37]. The replacement of the opal codon with an Arg codon in an infectious CHIKV clone from Sri Lanka did not adversely affect CHIKV replication in cultured human or insect cells, or in target tissues of C57BL/6J mice [37]. Nevertheless, compared to the WT virus, the opal-to-Arg mutation was associated with decreased virulence in vivo, causing significantly decreased footpad swelling, delayed production of proinflammatory cytokines and chemokines and reduced inflammatory cell infiltration, including CD4^+^ T cells and NK cells, within the joint of infected mice. In that study, CHIKV-induced disease in mice was associated with the presence of the opal termination codon and independent from viral replication. This effect is, however, not conserved in all alphaviruses, as can be seen from experiments with different SFV strains.

Introducing an opal stop codon into SFV4 lead to dramatic attenuation when tested in adult BALB/c mice [38]. For the majority of SFV4-opal infected mice, the virus titer in the brain was even lower than for those infected with the avirulent SFV A7(74) strain. Remarkably, the opal codon of recombinant SFV4-opal that was isolated from the brain of two moribund mice, associated with high brain virus titers, was found to have reverted to an Arg or Trp codon, highlighting the important role of the Arg codon in virulence regulation for SFV4. In contrast to SFV4, SFV A7(74) does contain an opal termination codon. Infections with SFV A7(74) are avirulent in adult mice, as mentioned above but a recombinant A7(74) virus, having an Arg in place of the opal stop codon (rA774-arg), as in SFV4, shows increased pathogenicity, despite still being far less virulent than SFV4 or when the entire nsP3 was exchanged [38,39]. Tuittila and Hinkkanen subsequently analyzed the virulence determining property of nsP3 more thoroughly, based on single amino acid residues that differ between SFV A7(74) and SFV4 [39]. Specific mutations were tested in the context of rA774-V4del-arg, an SFV A7(74) derivative containing the Arg stop codon, as well as a 21-nucleotide insertion, naturally present in SFV4 but absent in SFV A7(74). This insertion does not influence virulence [38] but was applied to allow for potential interactions with mutated residues. The authors were able to pinpoint several combinations of two nsP3 amino acid mutations that fully reconstitute neurovirulence of rA774-V4del-arg. These residues were found at different locations of nsP3, suggesting an important role for the structure of nsP3 during replication.

## 3. A Degradation Signal at the Extreme C-Terminus of nsP3 Regulates Its Expression

During infection, nsP3 is usually found in complex with other nsPs or involved in interactions with host factors and thus was considered to be a rather stable protein until Varjak and colleagues discovered a degradation signal at the C-terminal region of SFV and SINV nsP3 [40]. Rapid degradation was observed when nsP3 was individually expressed, while the protein was significantly stabilized when expressed in the form of polyprotein nsP123. Moreover, rapid degradation of nsP3 appeared only at the early stage of viral infection, probably due to the liberation of subpopulation of nsP3 from non-structural polyproteins. The degradation signal was narrowed down to the last 6-10 residues at the C-terminus of SFV nsP3 and the last 36 residues at the C-terminus of SINV nsP3. When fused to reporter proteins EGFP and luciferase, both degradation signals were shown to be functionally effective. The roles of the degradation signal remain unknown but it is likely that it contributes to achieving optimal stoichiometry of non-structural proteins, which is well organized and regulated during viral infection. The amount of nsP4 was increased when two copies of nsP3 were expressed from a recombinant SFV6 [41], suggesting that the degradation of nsP3 in WT SFV infection could contribute to regulating nsP4 levels. Considering both SFV and SINV contain similar degradation signals [40] but that SFV4 does not encode an opal stop codon at the nsP3/4 junction, it is reasonable to assume that the presence of the degradation signal is unlikely related to that of the opal stop codon. In contrast to SFV, SINV expresses two isoforms of nsP3, with the effective degradation signal only present in the long isoform. Thus, Varjak and colleagues speculated that for alphaviruses with two isoforms of nsP3, the degradation signal is perhaps involved in the regulation of ratio of isoforms. Apart from SFV, MAYV nsP3 contains the same last 6 residues of the degradation signal at its C-terminus and very similar sets of C-terminal residues are also present in RRV nsP3 but their function as degradation signals needs further validation.

## 4. Localization of nsP3 in Infected Cells

In cell fractionation studies, the majority of CHIKV nsP3 was found in the cytosolic S15 fraction [42], while the majority of SFV nsP3 was found in association with nsP1 and nsP4 in the membrane pellet (P15) fraction of infected BHK-21 cells [43]. The RNA polymerase activity of SFV replication complexes was found exclusively in the P15 fraction [44] and remains strictly membrane-associated [45]. In infected cells, nsP3 can be detected on the cytoplasmic surface of viral replication complexes, that first appear at the plasma membrane (PM) and for some alphaviruses later also in form of CPVs in the perinuclear area [15,46,47]. While a fraction of processed, mature nsP3 remains associated with the other nsPs as an integral part of the viral replication complex, a subpopulation of nsP3 is liberated and forms large cytoplasmic aggregates [43,48]. The relative levels of nsP3 associated with active RNA replication complexes and that in the larger cytoplasmic aggregates varies for the different alphaviruses, for example most of the nsP3 staining in SFV-infected cells colocalizes with dsRNA, while a much smaller fraction does so in CHIKV-infected cells, with most staining present in larger cytoplasmic aggregates. The larger aggregates also form when nsP3 is expressed individually [21,49,50] and their appearance is likely influenced by interactions with host proteins. Studies with SINV and VEEV have shown that the HVD of nsP3 influences the composition as well as morphology of nsP3-containing cytoplasmic complexes, likely due to different sets of host protein interactions influencing aggregate structure [51]. The number of cytoplasmic nsP3 aggregates is reduced in mutants that fail to bind the host protein Ras-GTPase-activating protein (SH3-domain)-binding protein (G3BP), for example references [40,52,53]. The presence of cytoplasmic nsP3 aggregates, separated from replication complexes, suggests that nsP3 fulfils additional functions, independent of viral genome replication.

The stoichiometry and localization of the individual nsPs within the spherules is not fully understood. Likewise, although we know of several host proteins associated with ns proteins, whether they localize to spherules or their orientation therein is largely unknown. A study mapping interactions between individual CHIKV nsPs, detected an interaction between nsP3 and nsP1, which in turn interacted with nsP2 and nsP4 [54]. It was proposed that nsP1 and nsP3 occupy much of the space in the narrow spherule neck, while nsP2 and nsP4 remain internal. Although highly speculative, such localization of nsP3 might allow its largely unstructured hypervariable domain (see below) to extend out of the spherule allowing those host protein interactions that are associated with the spherules to be maintained exterior to the replication complex itself.

## 5. NsP3 as a Vector Specificity Determinant

Alphaviruses are arboviruses and need to replicate in vertebrate as well as in invertebrate hosts. In this context, nsP3 serves a critical function in determining vector specificity, as shown by experiments with the Old World alphaviruses CHIKV and ONNV in mosquitoes [55]. ONNV is the only known alphavirus that is transmitted by anopheline mosquitoes, while CHIKV primarily infects *Aedes* species. *Anopheles gambiae* mosquitoes are naturally refractory to CHIKV WT infection but become susceptible when exposed to chimeric CHIKV, expressing ONNV nsP3. This observation supports previous suggestions that nsP3 may exert host cell-dependent functions, presumably through specific protein-protein interactions [56]. In addition, a recent report describing RNAi suppressor activity for nsP3 suggest very different functions for nsP3 in insect and mammalian cells [57].

## 6. NsP3 Is a Major Determinant of Neurovirulence for Some Alphaviruses

Some strains of SFV are more neurovirulent than others and nsP3 has been found to be a major contributing factor to this [38,39]. In contrast to the neurovirulent strains SFV4 and SFV6 (a fully virulent consensus clone of the SFV L10 strain), the SFV A7(74) strain is avirulent in adult mice. A modified A7(74) strain, in which the nsP3 was replaced with the nsP3 of SFV4 or SFV6, showed lethal neurovirulence [38,41]. Even a recombinant SFV A7(74) expressing an additional heterologous copy of SFV6 nsP3 was significantly more virulent [41]. Thus, with the insertion of SFV6 nsP3, a dominant neurovirulent phenotype can be transferred to SFV A7(74). Interestingly, a reciprocal change, with SFV6 nsP3 replaced by nsP3 of SFV A7(74), still resulted in a virulent phenotype, killing infected BALB/c mice with similar kinetics as parental SFV6. These experiments show that SFV virulence is associated with nsP3 but as for SFV6, it is not the exclusive determinant. An 18-amio acid deletion in the C-terminal domain of SINV nsP3 was also found to be one of several neurovirulence determinants in the adult mouse [58]. It should be noted that although nsP3 has been implicated in neurovirulence of the Old World alphaviruses, the same is not true for the New World viruses, with the main determinant being the structural proteins, particularly E2 [59].

## 7. NsP3 Is a Hub for Multiple Host Protein Interactions

The mature nsP3 protein varies in size from 469 to 570 residues in different alphaviruses. It is a modular protein, organized in three domains, the N-terminal macro domain, the central zinc-binding domain and the C-terminal hypervariable domain (Figure 1). The current state of knowledge of these three domains and their associated host proteins are described in detail in subsequent sections. Several studies have used mass spectrometry, phage display or other screening methods to identify a profusion of host proteins as nsP3 interactors [18,46,60,61,62]. Other studies have identified interactors through hypothesis-driven investigations [63,64,65]. We present a collated list of cellular proteins that have been reported to interact with alphavirus nsP3 (Table 1). Many prominent interactions have been confirmed in other studies and some have been mapped to motifs, mostly in the HVD [62,63,66,67,68]. Host protein interactions which have been well validated, mapped to a particular region of nsP3 or for which a clear role in the viral life cycle is known, are described in detail below. There is a picture emerging of nsP3 as a hub for interactions with multiple cellular proteins, directing modulation of multiple cellular pathways during infection. Presented in the table are 92 and 32 interactors for Old World and New World alphavirus nsP3, respectively. Most of these are unique for one or the other, supporting the previous observation that Old and New World alphaviruses form distinct sets of host proteins interactions [51]. Nevertheless, that no fewer than 16 interactions are shared, suggests perhaps a more significant overlap than previously considered.

## 8. The Macro Domain

The N-terminal macro domain, first described as X-domain [75], is conserved among alphaviruses and homologous domains can also be found in proteins of other positive-strand RNA viruses (including rubella virus, some coronaviruses and hepatitis E virus), as well as some bacterial and cellular proteins [76,77]. Solution NMR spectroscopy of the CHIKV macro domain revealed a mixed α/β/α topology with 4 α-helices and 6 β-strands [78]. This is in agreement with the NMR structures obtained for the macro domains of MAYV [79] and VEEV [80] and the crystal structures of the CHIKV & VEEV macro domains [81].

Several studies have shown that viral macro domains bind ADP-ribose, dephosphorylate ADP-ribose-1′′-phosphate and have de-ADP-ribosylating activity (reviewed in [82]). In order to compare the nsP3 macro domains of Old and New World alphaviruses, Malet et al. analyzed the macro domains of CHIKV and VEEV biochemically and structurally [81]. The macro domains of both viruses bind to monomeric ADP-ribose (MAR) and poly(ADP-ribose) (PAR) and possess ADP-ribose 1′′ phosphate phosphatase activity, converting ADP-ribose-1′′-phosphate to ADP-ribose. The macro domain of SFV nsP3 also binds PAR [81,83,84] but in contrast to nsP3 of CHIKV and VEEV, binding to ADP-ribose monomers could not be detected [83] and ADP-ribose 1′′ phosphate phosphatase activity was at the limit of detection [84]. This might indicate that the alphavirus macro domains share some functional properties but are not identical.

More recently, it was demonstrated that viral macro domains, such as for VEEV nsP3, remove MAR and PAR from MARylated and PARylated substrates, respectively [85]. The de-MARylation activity was subsequently also confirmed for the macro domains of CHIKV, SINV and ONNV but removal of PAR chains, in contrast, was found to be considerably less efficient for these viruses [86]. In yet another recent study about its enzymatic activity, McPherson and colleagues showed that the macro domain of CHIKV nsP3 actively hydrolyzed MAR from modified aspartate and glutamate residues but not from lysines [87]. The hydrolase activity of the macro domain relies on its affinity to ADP-ribose and was dramatically inhibited and CHIKV recombinants were nonviable when the ADP-ribose binding of nsP3 macro domain was abolished. Moreover, mutations affecting the ADP-ribose affinity and/or hydrolase activity of the macro domain of CHIKV nsP3 resulted in attenuated viral growth in cell culture and reduced virulence in mice to varied extents, highlighting the important role of this domain [87]. In agreement with this observation, mutations introduced in the putative ADP-ribose binding site of SINV nsP3 macro domain resulted in impaired viral replication in cultured neurons and attenuated virulence in mice [64]. Despite their impact, these mutations did not alter PAR binding. Whether their affect was instead due to an altered enzymatic activity remains to be tested. Meanwhile, several residues that play crucial roles for the function of viral macro domains are known, as reviewed in [82].

In addition to the described function, other potential roles for alphaviral macro domains should also be considered. For example, the presence and proper location of the domain has been shown to be involved in the cleavage of nsP2/3 in SFV, probably by serving as the recognition site of protease nsP2 [88]. Furthermore, binding of ssRNA was also observed, as exemplified by the CHIKV nsP3 macro domain, and an early study showed that mutation at a single residue glycine 68 in SINV nsP3, which was predicted to be residing in an active site, blocked the majority of nsP3 phosphorylation and the synthesis of viral minus sense RNA [89].

The expression of several cellular ADP-ribosyltransferases is stimulated by interferon [86,90]. It has therefore been hypothesized that ADP-ribosylation is a component of the antiviral response and may be counteracted by the de-ADP-ribosylation activity of viral macro domains. However, further studies are required to fully understand the functional relevance of MAR and PAR binding and removal for viral replication. Given the conservation of the macro domain in many positive-strand RNA viruses [76,77], blockage of its hydrolase activity has been proposed to be a direction for the development of broad antiviral therapy [87].

## 9. The Central Alphavirus-Unique Domain (AUD) Is a Zinc-Binding Domain (ZBD)

The central part consists of a conserved alphavirus-unique domain (AUD), with undefined but important roles in RNA replication. Viable mutants in this domain were shown to exhibit defects in early events in RNA replication, likely the formation of the replication complex for minus-strand synthesis [25,26]. Since recently the domain is also referred to as the zinc-binding domain (ZBD) [91]. Four cysteine residues within the central domain, that are absolutely conserved among alphaviruses, coordinate a zinc ion. Infectious center and plaque assays of Cys → Ala mutants showed that each Cys residue is individually essential for virus replication. In addition, a patch of basic amino acids close to the zinc-binding site has been proposed to be involved in RNA binding. Despite these observations, the functional significance of this domain is unclear. The domain is conserved among alphaviruses and has a distinct structure. The most comprehensive structure of nsP3 available is that of an uncleaved SINV nsP23 precursor which includes the protease and methyltransferase-like (MT-like) domains of nsP2, as well as the N-terminal macro and the central ZBD domain of nsP3, only lacking the HVD [91]. According to this structure, the P2/3 cleavage site resides at a narrow cleft formed by the MT-like domain of nsP2 and the nsP3 macro domain and is 40 Å away from the nsP2 protease active site, which supports that the P2/3 processing junction is cleaved in *trans*. The nsP3 macro domain and ZBD form a ring-like structure. Both domains share an interface with 570 Å of buried surface area and are connected by a linker region. The ring-like structure of nsP3 forms an extensive charged interface with nsP2 and encircles its MT-like domain. The importance of interface formation is highlighted by the fact that several mutations within nsP2, which were identified in non-cytopathic viruses, map to this interface and lie in close proximity to the nsP3 linker. Site-directed mutagenesis of the nsP3 linker region confirmed that the P23 interface is important for RNA infectivity and in addition also influences P23 processing efficiency.

## 10. The Hypervariable Domain (HVD)

**Figure 2:**
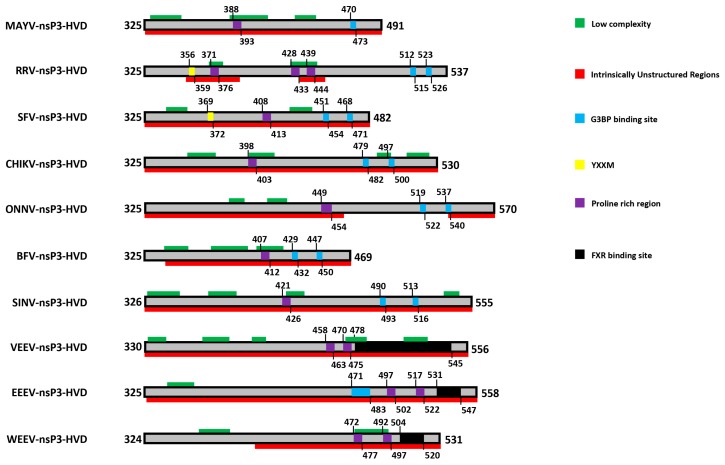
Prominent features in HVD of various alphaviruses. Selected features are displayed with different colors as indicated on the right. For details please see text. Prediction of low complexity regions was carried out using the SEG program [98] and intrinsically unstructured regions were predicted using http://anchor.enzim.hu/ [99]. The Uniprot entries of nsP3 sequences used for the analyses are: MAYV (Q8QZ73), RRV (P13887), SFV (P08411), CHIKV (Q8JUX6), ONNV (Q8QZ73), BFV (P87515), SINV (P03317), VEEV (P36328), EEEV (Q4QXJ8), WEEV (P13896).

In contrast to the rest of the protein, the C-terminal domain of alphaviral nsP3 shows high variability in sequence and length even between closely related alphaviruses and has therefore been termed the hypervariable domain (HVD). The HVD of all alphaviruses analyzed is largely of low complexity or intrinsically disordered, without any major predicted secondary structure (Figure 2). Despite being largely non-conserved between alphaviruses [31], it contains a number of features common to many of the members of the family that appear to be mediating multiple host protein interactions. Features in the HVD include a hyperphosphorylated region (mapped to residues 319 to 368 in SFV) [49,56,92] and a proline rich region (in varying positions in different viruses) [62] as well as several repeated elements in the C-terminal region [31,93,94] (Figure 2). Unique duplicates of 36 nucleotides (corresponding amino acids: HADTVSLDSTVS/L) were found to be present in all RRV strains isolated from and after 1979 but absent in any earlier isolate or other alphaviruses [95]. The adaptation of the duplication, interestingly, corresponds to the increase of annual average infected cases of RRV, from around 500 in 1980 to around 5000 at present. However, there is no described mechanistic benefit for the virus, even though the duplicates seem to confer upon the viral genome a more stable RNA structure with a stem loop based on the predicted secondary structure of the repeat-containing region of RRV nsP3 [95]. Similarly, nsP3 of VEEV possesses two repeats of 102 nucleotides, that were also predicted to form a stable stem loop structure [96]. Apart from the 36 nucleotide duplication described above, other repeats were also observed in the HVD of RRV nsP3, among those, one type of repeats PXPXPR was later identified as the binding site for host factor amphiphysins [62], as discussed below. Furthermore, close to the C-terminus, two repeat sequence elements containing FGDF-like motifs are conserved among Old World alphaviruses [40,68]. Similarly, a distinct repeat sequence is also present in different strains of the New World alphavirus VEEV [93,97]. These repeat sequences are binding sites for specific host proteins, G3BP and FXR proteins for nsP3 of Old and New World alphaviruses, respectively and will be the subject of further discussion below.

The HVD of nsP3 is essential for alphavirus replication but several studies showed that this domain tolerates deletions and insertions to some extent [48,56,96,100]. This has been widely exploited for the generation of nsP3 fusion proteins, used successfully in immunofluorescence and immunoprecipitation experiments to follow the subcellular distribution of the protein and to discover novel interaction partners [13,18,46,48,101]. On the other hand, the HVD also contains critical single residues that, once mutated, have a strong impact on viral replication efficiency [68,102].

The hypervariable domain of nsP3 is to a large extent intrinsically disordered (Figure 2) and such protein regions are known to facilitate binding to multiple partners, often involving a disorder-to-order transition [103]. Several interactions have been described in the HVD of various alphaviruses but the location of the binding site is not yet known. The PAR polymerase-1 (PARP-1) interacts with the C-terminal HVD of SINV nsP3 in NSC34 neuronal cells and is transiently recruited to viral replication complexes [64]. Both the phosphorylation of nsP3 and the enzymatic activity of PARP-1 to synthesize PAR were found to be dispensable for this interaction, suggesting that the interaction is not mediated by PAR. In fact, inhibition of PARP-1 activity did not affect virus replication, suggesting that PARP-1 might exert its functional role through direct protein interactions. SINV replication was not affected by PARP-1 deficiency but this deficiency resulted in delayed apoptosis [104]. On the other hand, SINV infection leads to PARP-1 activation and subsequently the synthesis of PAR [104]. Whether the interaction of PARP-1 at the HVD has any functional relevance for the activity of the macro domain remains to be determined.

A mass spectrometry approach identified several host proteins interacting with VEEV nsP3, among them the DEAD-box RNA helicases DDX1 and DDX3 and others (described below) [70]. Depletion of DDX1 and DDX3 by siRNA reduces viral titers. DDX3 is known to associate with factors of the host translation machinery, subsequent investigations showed that the nsP3-DDX3 complex also recruits eIF4A, eIF4G and PABP. VEEV nsP3 might thus promote translation initiation of viral mRNA. That so many host protein interactions occur in the context of the unstructured HVD may imply its involvement in the adaptation to new hosts in for example the arthropod to mammal transition.

## 11. The HVD Is Phosphorylated

NsP3 is the only non-structural protein found to be phosphorylated [43,105]. In SFV-infected BHK cells nsP3 was found to contain phosphoserine and phosphothreonine residues but phosphotyrosine was not detected [43,92]. Approximately double the amount of phosphoserine was detected, relative to phosphothreonine [43]. Peränen and colleagues speculated that the phosphorylation might serve a regulatory function during RNA synthesis and pointed out that the fraction of nsP3 that is associated with RNA polymerase activity in the P15/membrane fraction is more heavily phosphorylated than that in the S15/cytosolic fraction [43]. The phosphorylation sites of SFV nsP3 were mapped by mass spectrometry and the majority of residues were located within the N-terminal 50 residues of the HVD domain, including S320, S327, S332 and S350 and 7–12 residues between amino acids G338 to K415 [49,92]. The majority of the phosphothreonine signal was provided by T344 and T345. Deletion of residues 319–368 in the mutant SFV-Δ50 resulted in undetectable levels of phosphorylation and in reduced levels of viral RNA synthesis at early stages of infection in cell culture but production of virus particles is similar to WT SFV [49]. In mice, however, infection with SFV-Δ50 was associated with greatly reduced neurovirulence, compared to WT SFV [49]. A study conducted with SINV nsP3 mutants indicated that phosphorylation was not essential for virus replication in chicken embryo fibroblasts but may be important for optimal replication depending on the host cell [56]. Other SINV mutants revealed that reduction in phosphorylation correlated with reduced minus strand RNA synthesis [89]. For VEEV, mutation of 53 potential phosphorylation sites within the HVD of nsP3, only had a minor effect on virus replication in BHK-21 cells but strongly impaired virus growth in mosquito C7/10 cells [97]. The cellular kinases responsible for the phosphorylation of nsP3 have not been identified. For SINV, casein kinase II (CK II) has been suggested to contribute [105] but additional kinases are likely involved, for example protein kinase C (PKC) [92].

## 12. A YXXM Motif Directs Hyperactivation of the PI3K-Akt-mTOR Pathway

Alphaviruses have been known to activate the cellular pro-survival and anti-apoptotic phosphatidylinositol-3-kinase (PI3K)-Akt-mammalian target of rapamycin (mTOR) pathway, although to different extents [106,107,108,109]. For example, SFV infection induces rapid, strong and sustained activation of the pathway—termed hyperactivation—while weaker activation by CHIKV is detected only later in infection [106,109]. SINV was also shown to exhibit efficient replication in HEK293 cells in the presence of Akt/mTOR inhibitors and even to suppress activation of the pathway at late stages of infection [107]. Contributing to the murky picture was that a clear benefit of activation of the pathway was not understood.

Recently, a partial explanation for the mechanism of PI3K-Akt-mTOR activation by alphaviruses was provided by the discovery of a YXXM motif in the HVD of a subset of alphaviral nsP3 sequences [63]. In SFV, the motif is immediately downstream of the 50 amino acids deleted in the SFV-Δ50 mutant. In addition to SFV, viruses carrying this motif include RRV, Getah, Sagiyama and Middelburg alphaviruses and similar motifs have been found to activate the PI3K-Akt-mTOR pathway in other viral systems [110,111]. The motif works by mimicking activated growth factor receptors at the plasma membrane, leading to activation of the pathway. Inactive PI3K exists as a heterodimer of a regulatory subunit p85 and a catalytic subunit p110, with p85 serving as a stabilizer and inhibitor of the p110 subunit. In mammalian cells, there are three isoforms of the p110 subunit (α, β and δ) and two of the p85 regulatory subunit (α, β). Subunit p85 contains two src homology 2 (SH2) domains, which interact with phosphotyrosine in YXXM motifs on activated growth factor receptors [112]. As a result of this interaction, active p110 is released from the heterodimer and recruited to the plasma membrane, where it converts the plasma membrane lipid phosphatidylinositol-4,5-bisphosphate (PI(4,5)P_2_) to phosphatidylinositol-3,4,5-trisphosphate (PI(3,4,5)P3). Since both Akt and its kinase phosphoinositide-dependent kinase 1 (PDK1) contain a pleckstrin-homology (PH) domain for binding to PI(3,4,5)P3, they are recruited into close proximity at the plasma membrane, facilitating the phosphorylation and activation of Akt.

The interaction between nsP3 and p85 was found to be dependent on both the Tyr and Met residues in the YXXM motif and also on the SH2 domains of p85 [63]. Replacement of the YXXM tyrosine by phenylalanine—which has the same structure as tyrosine except for the absence of the phosphorylatable –OH group—was enough to abolish the nsP3–p85 interaction and the PI3K–Akt hyperactivation phenotype. This suggests that the Tyr residue is phosphorylated although tyrosine phosphorylation of SFV nsP3 was not detected in biochemical studies [43,92] and our own observations. We propose that during infection, only a small subset of nsP3 molecules are phosphorylated on the Tyr residue in a transient manner. It remains to be determined which cellular kinase catalyzes phosphorylation of the tyrosine in SFV nsP3 and how its activity is regulated during infection. The interaction was also dependent on localization to the PM. In infected cells, nsP3 is recruited along with the other non-structural proteins to the PM via membrane association of nsP1 [19,21] and the interaction occurs there [63]. Expression of nsP3 in the absence of other viral sequences did not allow the interaction with p85 to occur in a detectable manner. However, when expressed nsP3 was targeted to membrane compartments by fusion to the myristoylation and palmitoylation signal of murine Lyn kinase, the interaction was readily detected [63,109]. This result underlines the importance of appropriate cellular localization for detecting cellular interactors.

There are several known effects of PI3K-Akt-mTOR activation on the SFV and RRV life cycle. Perhaps the most striking effect is the noticeable relocalization of viral RNA replication complexes from PM adjacent in the early stages of replication to the more internal localization [13,63,109]. This relocalization is dependent on the PI3K-Akt-mTOR hyperactivation: in cells infected with CHIKV or with SFV or RRV mutants which do not hyperactive the pathway, the dsRNA signal remains strong at the PM and cellular periphery. However, CHIKV RCs were efficiently internalized (and the PI3K-Akt-mTOR pathway was hyperactivated) in a mutant in which the HVD was switched for that of SFV [109]. Despite our recent understanding of this mechanism, we are still unable to explain why internalization of spherules to form internal CPVs is beneficial to these viruses.

Full activation of Akt results in phosphorylation of many substrates related to cell metabolism and survival. The mechanistic target of rapamycin (mTOR) in the mTOR–raptor complex (mTORC1) is one of the main downstream substrates of Akt. Activation of mTORC1 leads to phosphorylation of ribosomal subunit S6 and de-phosphorylation of 4EBP1, the inhibitor of the cap-binding protein eIF4E, thus promoting protein synthesis and keeping cells in a proliferative state. Thus, activation of mTOR complex in SFV- and RRV-infected cells, might contribute to the high and sustained levels of viral protein synthesis late into infection. Concomitant with that, Akt activation in SFV-infected cells was also shown to cause a shift in glucose metabolism towards glycolysis and the pentose phosphate pathway (PPP) [63]. This results in increase in synthesis of the precursors of nucleotides and fatty acids. Importantly the kinetics of this shift coincided with efficient viral replication suggesting the importance of this metabolic shift for virus production. Moreover, treatment of infected cells with inhibitors of glycolysis and the PPP lead to reduced viral titers. These observations suggest the intriguing possibility that pathogenic RRV infection could be treated with Akt inhibitors, of which there are several in clinical trials for their anti-cancer properties.

## 13. A Proline-Rich Region within the HVD Mediates Binding to Amphiphysin Proteins

The HVD of nsP3 of all alphaviruses contain proline-rich regions. For several alphaviruses, such as SFV, SINV and CHIKV this region includes the motif “P[I/V][P/A]PPR” but some viruses, SFV in particular, have extended polyproline stretches. PxxP motifs have been shown to bind Src homology 3 (SH3)-domain containing proteins. The SH3-domain is a small domain, typically involved in protein: protein interactions and found in dozens of proteins including those involved in several signaling pathways and cytoskeleton regulation [113,114]. Indeed, an SH3 bacteriophage display library was used to show that the SH3 domain of amphiphysin-1 and Bin1/amphiphysin-2 bind to the “P[I/V][P/A]PPR” motifs in SFV, CHIKV and SINV nsP3 [62]. An interaction between Bin1/amphiphysin-2 and the HVD of SINV nsP3 has also been shown in another study [67]. A proline-rich region, including a “P[I/V][P/A]PPR” motif can also be found in the C-terminal part of hepatitis C virus (HCV) NS5A protein. HCV NS5A was found to bind Bin1/amphiphysin-2 but deletion of the C-terminal part (aa 278–447) completely abrogated this interaction [115]. More precisely, proline-to-alanine mutation showed that the proline-rich region mediates complex formation with the SH3 domain of Bin1/amphiphysin-2. For SFV and SINV, disruption of the nsP3-amphiphysin interaction by either mutating nsP3 or knockdown of amphiphysin resulted in impaired RNA replication [31]. In addition, mutant SFV which does not interact with amphiphysin causes decreased neuro-pathogenicity in infected mice. As amphiphysins can induce membrane curvature, it has been speculated that they play a role in the formation of spherules but this has yet to be confirmed. In another study, the structure of the SH3 domain of Bin1/amphiphysin-2 was solved in complex with a CHIKV nsP3 peptide using solution state NMR spectroscopy [69]. Characterization of the binding dynamics by isothermal titration calorimetry (ITC) revealed that the CHIKV nsP3 peptide has an unusually high affinity for the Bin1/amphiphysin-2 SH3 domain in comparison to cellular ligands, such as dynamin. A peptide derived from SFV nsP3 also showed a relatively high affinity, albeit almost ten-fold lower than the CHIKV nsP3 peptide. Through subsequent mutation analysis combined with NMR and ITC experiments, the authors suggest a viral high-affinity amphiphysin binding motif with the consensus sequence P[I/V][P/A]PPR[R/K/P][R/K][R/K]. Electrostatic interactions between a negatively charged binding surface on the SH3 domain and several positively charged residues in the viral peptide ligands account for the high affinity. In addition to amphiphysins, the phage screen also identified the SH3 domains of the adapter proteins CMS/CD2AP, CIN85 and SASH1 as potential nsP3 binding sites [62] of which, CD2AP was also found to associate with VEEV nsP3 [67]. Apart from the amphiphysins, it is possible that other SH3-domain containing proteins are recruited to the polyproline motifs during alphavirus infection.

## 14. The HVD Contains Repeat Motifs Which Direct Binding to Host Stress Granule Proteins

In order to identify nsP3-mediated host protein interactions, Cristea et al. used mutant SINV encoding green fluorescent protein (GFP) inserted between residues 388 and 389 of nsP3 [46,48]. Immunoaffinity purification and mass spectrometry analysis identified 35 host factors interacting specifically with nsP3-GFP, including G3BP-1 and -2, heterogeneous nuclear ribonucleoprotein (hnRNP) A1, A3, A2/B1, G and tyrosine 3-monooxygenase/tryptophan 5-monooxygenase activation proteins ε, ζ and η (14-3-3 ε, ζ and η). While the nsP3:G3BP interaction appeared consistent throughout the course of infection, the associations with hnRNPs and 14-3-3 proteins were temporally regulated and found at early (2–4 hpi) and late times (6–10 hpi) of infection, respectively. It has been speculated that a change in the phosphorylation state of nsP3 is responsible for the time-dependent interaction but whether the phosphorylation of nsP3 changes during the course of infection remains to be investigated.

The nsP3-G3BP interaction has meanwhile been confirmed in several studies [46,60,66,67,68,116] and the binding to G3BP is the best characterized nsP3-host interaction for alphaviruses. G3BP is a multifunctional RNA-binding protein and is expressed in three isoforms, G3BP-1 and two splice variants G3BP-2a and G3BP-2b (here collectively referred to as G3BP). It has a well-described role as a stress granule (SG) nucleating protein [117,118]. SGs are dynamic aggregates of stalled translation preinitiation complexes that assemble in response to cellular stresses, such as heat and cold shock, oxidative stress and virus infection [119,120]. G3BP is essential for SG formation triggered by phosphorylation of eukaryotic initiation factor 2α (eIF2α) or inhibition of eIF4A [118]. Early in infection, SFV infection causes the formation of SGs via the detection of dsRNA replication intermediates by protein kinase R (PKR) leading to the phosphorylation of eIF2α [121]. As the infection cycle progresses SGs are disassembled [53,68,121]. For Old World alphaviruses, the mechanism of SG disassembly involves the sequestration of G3BP by nsP3. This has been investigated in detail for the model virus SFV which shall be described hereafter but analogous mechanisms likely also apply to other Old-World alphaviruses.

NsP3 binds to the nuclear transport factor 2 (NTF2)-like domain of G3BP [46,52] via its two Phe-Gly-Asp-Phe (FGDF) motifs (Figure 2), found close to the C-termini of the HVD of Old World alphavirus nsP3 [68]. Mutagenesis experiments revealed that both Phe residues and the Gly residue in each motif are essential for the interaction with G3BP. A 3-dimensional structure of the NTF2-like domain of G3BP in complex with an nsP3-derived 25-mer peptide revealed that the FGDF motif-containing peptide occupies a hydrophobic groove in the NTF2-like domain with both phenylalanine residues inserted into pockets in the groove. The necessity of the Gly residue is explained by a kink in the peptide backbone at that position which allows the Phe residues to take their positions [102]. Upon binding by nsP3, G3BP is sequestered to viral replication complexes and other sites of viral protein aggregation [52,53]. As nsP3 levels rise, G3BP sequestration leads to the disassembly of SGs. Mutant SFV, which does not bind G3BP (SFV-F3A) shows a prolonged SG response and virus growth is attenuated in cell culture [53,68]. However, this mutant replicates to levels similar to WT virus in cells that do not form SGs (eIF2α-AA MEF cells) suggesting that in MEFs, the primary function of the SFV nsP3-mediated G3BP sequestration lies in the inhibition of SG formation.

It has been hypothesized that nsP3 evolved to contain two G3BP-binding motifs to ensure rapid neutralization of G3BP and subsequent SG disassembly. But structural analysis of G3BP in complex with nsP3 suggested an additional biological function. Schulte and Liu et al. reported a high-resolution crystal structure of the NTF2-like domain of G3BP1 (residues 1–139) in complex with a SFV nsP3-derived peptide (residues 449–473) containing both of the conserved “FGDF” sequence elements [102]. In agreement with previous observations [122,123], the NTF2-like domain forms dimers and the FGDF motif occupies a hydrophobic groove between alpha helices on the surface of the NTF2-like domain of G3BP. Each of the two FGDF motifs in the nsP3 peptide was found to bind to a G3BP monomer on separate dimers. Thus, it is likely that nsP3 interconnects G3BP dimers and induces the formation of a poly-complex. As SFV mutants with either only one G3BP-binding motif (SFV F3A_N_ or SFV F3A_C_) or none (SFV F3A_NC_) are equally attenuated, both motifs are required for efficient virus replication [53,102]. This suggests an important role for the nsP3:G3BP poly-complex, which can only form if both FGDF motifs are present. It has been proposed that the nsP3-G3BP oligomers could function to stabilize viral replication complexes, by tying them together and thereby inducing high local concentrations of viral factors and in addition forming a protective layer against cellular antiviral mechanisms [102].

Similar to the model virus SFV, nsP3 of the human pathogen CHIKV also comprises two G3BP-binding motifs, suggesting an identical mechanism for the recruitment of G3BP [52,102]. In contrast to SFV, which is attenuated but still replicates to detectable levels in the absence of G3BP, CHIKV mutants that do not bind G3BP are essentially non-viable [67,102]. siRNA-mediated depletion of G3BP proteins in CHIKV infected cells results in severely impaired production of viral RNA [124] and CHIKV is non-viable in CRISPR/Cas9 G3BP knockout cells [67]. This suggests that G3BP exerts a pro-viral role during CHIKV replication and that SG disassembly is in fact not the only function of the nsP3-G3BP interaction. The CHIKV nsP3-G3BP interaction is also conserved in the invertebrate host. The homologue of G3BP in the insect was termed Rasputin (Rin), after its putative role in Ras signaling [125]. Full length *Drosophila melanogaster* Rin shares 40% identity and 60% homology on the amino acid level with human G3BP1 and G3BP2, with higher conservation and very similar structure in the NTF2-like domains [126]. *Aedes albopictus* Rasputin, also well conserved, was found to interact with CHIKV nsP3 in a similar manner as in the mammalian host [71]. Knockdown of Rin in vivo resulted in reduced infection rates as well as reduced viral titers, suggesting the importance of this interaction for viral replication also in the mosquito vector.

Old and New World alphaviruses are geographically separated and have evolved independently to interact with different sets of host factors. Accordingly, different nsP3-specific complexes form when cells are infected with either VEEV (New World) or SINV (Old World) [51]. The HVD of New World alphavirus nsP3, such as VEEV, does not contain FGDF motifs and do not bind G3BP [51,67]. Nevertheless, VEEV nsP3 recruits another set of SG-related proteins via its HVD, members of the FXR protein family (Fragile X mental retardation protein 1 (FMR1), Fragile X mental retardation syndrome-related protein (FXR) 1 and 2, collectively referred to herein as FXR) [67]. CRISPR/Cas9 knockout of FXR genes strongly reduced VEEV replication efficiencies. Thus, Old World and New World alphaviruses recruit distinct sets of cellular proteins, which however share common properties and likely fulfil similar functions during alphavirus infection. Both FXR and G3BP proteins are critical proteins in SG assembly and contain RNA-binding domains, which might explain their similar functions in New and Old World alphaviral replication.

For alphaviruses, it seems that G3BP and FXR protein families are critical interaction partners exclusive for Old World and New World alphaviruses respectively. However, Frolov et al. revealed that HVD of EEEV nsP3 mediated the interaction with both protein families and, at early stage of infection, recruited them into viral replication complexes independently [66]. The binding motifs in EEEV nsP3 were revealed as amino acid residues of 471–483 for G3BP and 531–543 for FXR. In comparison, the binding site for G3BP in EEEV nsP3 is very different from that of nsP3 of Old World alphaviruses [46,52,66]; while the FXR binding site only shares some similarities with that of VEEV nsP3 [51,66,67]. The interaction sites for EEEV nsP3 are the NTF2 domain of G3BP, same as previously described [46,52] and the agenet-like domain of FXR [66]. Moreover, disruption of the interaction between EEEV nsP3 with either G3BPs or FXRs brought subtle effect on viral growth, while only the simultaneous disruption of both interactions significantly suppressed viral growth, indicating the functional redundancy of G3BPs and FXRs for EEEV infection. But this may contribute to the efficient replication and high virulence of EEEV, as suggested by the authors [66]. Chimeric VEEV containing G3BP-binding sites in place of those for FXR was capable of efficient replication even in the absence of FXR, while chimeric CHIKV containing FXR-binding sites in place of the FGDF motifs, was nonviable [67]. This suggests that FXR are later developed host interaction factors than G3BP. Thus, EEEV could be seen as an intermediate during the evolution of alphaviruses, considering the exclusive use of G3BPs by Old World alphaviruses and FXRs by VEEV (New World alphavirus) [66]. Whether or how EEEV benefits from the ability to use both G3BPs and FXRs is unknown.

The nsP3-G3BP/FXR interaction has been subject to several studies and has been described in great molecular detail. However, some details of the mechanism by which these cellular RNA-binding SG-associated proteins exert their, in some cases, critical function remains to be shown. Nevertheless, both G3BPs and FXRs are apparently favorable targets for development of antiviral drugs.

## 15. Conclusions

For decades, nsP3 has been referred to as a protein of unknown function but with published data accumulating, the enigmatic protein is slowly revealing its secrets. A picture is emerging in which nsP3 exerts a crucial function in mediating multiple virus-host protein-protein interactions. Some of these have already been investigated in great detail, while others remain to be validated and their mechanism to be resolved. It is likely that more cellular protein interactions will be described in the coming years and a clearer picture will emerge of the role of nsP3 and its interactors in the alphaviral life cycle. As we learn more about the multiple linear motifs in the HVD, their host protein interaction and effects on the host cell biology, we can engineer alphavirus vectors with different properties for use in vaccine and oncolytic vector studies. The importance of the macro and zinc-binding domains will become clearer also and as conserved domains they could prove to be good targets for a more far-ranging anti-alphaviral pharmaceutical intervention. Importantly, as pharmacological treatments for alphavirus infections are still lacking, the numerous nsP3-mediated virus-host interactions offer many potential targets for interference.

## Figures and Tables

**Table 1:** Alphavirus nsP3-host interactions. The table summarizes cellular proteins reported to interact with alphavirus nsP3. Viruses and cell types involved in each study are indicated. For methodology please refer to the original publications. Cell types: BHK-21—Baby hamster kidney fibroblasts; C_7_10—*Aedes albopictus*; HEK293—Human embryonic kidney; 293T—HEK293 expressing SV40 large T antigen; 293FT—Fast-growing, highly transfectable clonal isolate derived of 293T; HeLa—Human cervix epitheloid carcinoma; MEF—Mouse embryonic fibroblasts; N2A—Mouse neuroblastoma; NIH 3T3—Mouse embryonic fibroblasts; NSC34—Mouse motor neuron-like hybrid; Rat2—*Rattus norvegicus* fibroblasts; Sf21—*Spodoptera frugiperda* ovarian cells; U87MG—Human astrocytoma cells.

Host Protein Interacting with nsP3	Cell Type	Virus	Ref.
14-3-3 z	C_7_10	SINV	[18]
14-3-3 β/α	BHK-21	SINV	[60]
14-3-3 γ	BHK-21	SINV	[60]
14-3-3 ε	Rat2, HEK293	SINV	[46]
	BHK-21	SINV	[60]
	C_7_10	SINV	[18]
14-3-3 ζ	Rat2, HEK293	SINV	[46]
14-3-3 ζ/δ	BHK-21	SINV	[60]
14-3-3 η	Rat2, HEK293	SINV	[46]
14-3-3 τ	BHK-21	SINV	[60]
Amphiphysin-1	In vitro, 293FT, N2A	SFV, SINV, CHIKV	[62]
Amphiphysin-2/BIN1—Bridging integrator 1	In vitro	CHIKV, SFV	[69]
	BHK-21	SINV	[67]
	In vitro, 293FT, HeLa	SFV, SINV, CHIKV	[62]
	NIH 3T3	CHIKV, SINV	[66]
B23.2	BHK-21	SINV	[60]
Beta-actin	BHK-21	SINV	[60]
Beta-tubulin	BHK-21	SINV	[60]
CAPZA1—F-actin-capping protein subunit alpha-1	BHK-21	VEEV	[67]
	NIH 3T3	EEEV, VEEV	[66]
CAPZA2—F-actin-capping protein subunit alpha-2	NIH 3T3	EEEV, VEEV	[66]
CAPZB—F-actin-capping protein subunit beta	BHK-21	VEEV	[67]
	NIH 3T3	EEEV, VEEV	[66]
CD2AP/CMS—CD2-associated protein	BHK-21	VEEV	[67]
	In vitro	SFV, SINV, CHIKV	[62]
	NIH 3T3	EEEV, VEEV, CHIKV	[66]
CKAP4—Cytoskeleton-associated protein 4	Rat2	SINV	[46]
CLTC—Clathrin heavy chain	NIH 3T3	EEEV	[66]
DDX1—DEAD box polypeptide 1	U87MG	VEEV	[70]
DDX3—DEAD/H box polypeptide 3	U87MG	VEEV	[70]
DDX5—DEAD box polypeptide 5	Rat2	SINV	[46]
	NIH 3T3	SINV	[66]
DDX6—DEAD box polypeptide 6	NIH 3T3	SINV	[66]
DDX17—DEAD box polypeptide 17	Rat2	SINV	[46]
	NIH 3T3	SINV	[66]
Desmin	BHK-21	SINV	[60]
DHX9—DEAD/H Box Polypeptide 9/ATP-dependent RNA helicase A	Rat2	SINV	[46]
DNAJC9—DnaJ heat shock protein family (Hsp40) member C9	NIH 3T3	CHIKV, SINV	[66]
Dnmt1—DNA (cytosine-5-)-methyltransferase 1	Rat2	SINV	[46]
eEF1A—Eukaryotic translation elongation factor 1A	BHK-21	SINV	[60]
eEF2—Eukaryotic translation elongation factor 2	Rat2	SINV	[46]
FHL2—Four and a half LIM domains protein 2	NIH 3T3	CHIKV	[66]
FMR1	BHK-21	VEEV	[67]
	NIH 3T3	EEEV, VEEV	[66]
FXR1	U87MG	VEEV	[70]
	BHK-21	VEEV	[67]
	BHK-21	SINV	[60]
	NIH 3T3	EEEV, VEEV	[66]
FXR2	BHK-21	VEEV	[67]
	NIH 3T3	EEEV, VEEV	[66]
G3BP-1/Rasputin	BHK-21	SINV	[60]
	Rat2, HEK293	SINV	[46]
	BHK-21	SINV	[67]
	BHK-21, MEF, HEK293	SFV	[53]
	In vitro	SFV	[52]
	BHK-21	SINV	[18]
	NIH 3T3	EEEV, CHIKV, SINV	[66]
	Sf21	CHIKV	[71]
	C_7_10	SINV	[18]
G3BP-2	BHK-21	SINV	[67]
	BHK-21, MEF	SFV	[53]
	BHK-21	SINV	[18]
	NIH 3T3	EEEV, CHIKV, SINV	[66]
	Rat2, HEK293	SINV	[46]
GRP78—78 kDa glucose-regulated protein	Rat2	SINV	[46]
	C_7_10	SINV	[18]
GSTM1—Glutathione S-transferase Mu 1	Rat2	SINV	[46]
HELQ—ATP-dependent DNA helicase Hel308	Rat2	SINV	[46]
HIST1H1C—Histone cluster 1 H1 family member C	NIH 3T3	CHIKV	[66]
hnRNP A0	BHK-21	SINV	[60]
hnRNP A1	Rat2	SINV	[46]
	BHK-21	SINV	[60]
hnRNP A1-like	U87MG	VEEV	[70]
hnRNP A2/B1	Rat2	SINV	[46]
	BHK-21	SINV	[60]
hnRNP A3	Rat2	SINV	[46]
	BHK-21	SINV	[60]
hnRNP C	HeLa	SFV	[61]
hnRNP G	Rat2	SINV	[46]
hnRNP K	HeLa	SFV	[61]
	HeLa, BHK-21	SINV	[72]
hnRNP M	HeLa	SFV	[61]
hnRNP U	BHK-21	SINV	[60]
HSC70—Heat shock cognate 71kDa protein	Rat2	SINV	[46]
	BHK-21	SINV	[60]
	BHK-21, C_7_10	SINV	[18]
HSP90—Heat shock protein 90	293T	CHIKV	[73]
HSPA1B—Heat shock protein 1B	NIH 3T3	EEEV, VEEV, CHIKV, SINV	[66]
IGF2BP1—Insulin-like growth factor 2 mRNA-binding protein 1	NIH 3T3	EEEV, SINV	[66]
IGF2BP2	NIH 3T3	EEEV, SINV	[66]
IGF2BP3	NIH 3T3	EEEV, VEEV, CHIKV, SINV	[66]
IKKβ—Inhibitor of nuclear factor kappa-B kinase subunit beta	U87MG	VEEV, WEEV	[65]
INTS6—Integrator Complex Subunit 6/RNA helicase HDB/DICE1	Rat2	SINV	[46]
JUN—Jun proto-oncogene	NIH 3T3	CHIKV	[66]
Lysophospholipase 1,2	C_7_10	SINV	[18]
MAP 1B—Microtubule-associated protein 1B	U87MG	VEEV	[70]
MYBBP1A—MYB binding protein 1A	NIH 3T3	CHIKV, SINV	[66]
MYH9—Myosin Heavy Chain 9	BHK-21	SINV	[60]
Myosin regulatory light chain	BHK-21	SINV	[60]
NAP1L1—Nucleosome assembly protein 1-like 1	NIH 3T3	CHIKV	[66]
NAP1L4—Nucleosome assembly protein 1-like 4	NIH 3T3	CHIKV	[66]
Nucleolin	Rat2	SINV	[46]
p85—PI3K subunit p85	HEK293, BHK-21	SFV, RRV	[63]
PARP-1—Poly(ADP-ribose) polymerase-1	NSC34, 293T	SINV	[64]
PCBP1/hnRNP E1—Poly(rC)-binding protein 1	HeLa	SFV	[61]
Peripherin 1	Rat2	SINV	[46]
PGAM5—PGAM family member 5, serine/threonine phosphatase	NIH 3T3	EEEV, VEEV, CHIKV	[66]
Plectin 1	BHK-21	SINV	[60]
	U87MG	VEEV	[70]
PSF—PTB-associated splicing factor	Rat2	SINV	[46]
RACK1/GNB2L1—Receptor of activated protein C kinase 1/Guanine nucleotide-binding protein subunit beta-2-like 1	Rat2	SINV	[46]
RDX—Radixin	NIH 3T3	CHIKV	[66]
Ribosomal protein L10	BHK-21	SINV	[60]
Ribosomal protein L10A	U87MG	VEEV	[70]
	BHK-21	SINV	[60]
	C_7_10	SINV	[18]
Ribosomal protein L15	Rat2	SINV	[46]
Ribosomal protein L18A	Rat2	SINV	[46]
Ribosomal protein L23A	C_7_10	SINV	[18]
Ribosomal protein L3	Rat2	SINV	[46]
Ribosomal protein L4	C_7_10	SINV	[18]
Ribosomal protein L5	BHK-21	SINV	[18]
Ribosomal protein L6	U87MG	VEEV	[70]
	BHK-21	SINV	[18]
Ribosomal protein L7	BHK-21	SINV	[60]
	C_7_10	SINV	[18]
Ribosomal protein L7A	BHK-21	SINV	[18]
Ribosomal protein L8	C_7_10	SINV	[18]
Ribosomal protein P0	U87MG	VEEV	[70]
Ribosomal protein S10	Rat2	SINV	[46]
Ribosomal protein S18	BHK-21	SINV	[60]
Ribosomal protein S3	Rat2	SINV	[46]
Ribosomal protein S8	U87MG	VEEV	[70]
	Rat2	SINV	[46]
Ribosomal protein S9	Rat2	SINV	[46]
	BHK-21	SINV	[60]
S100A4—S100 calcium binding protein A4	NIH 3T3	EEEV, VEEV	[66]
SASH1—SAM and SH3 domain-containing protein 1	In vitro	SFV, SINV, CHIKV	[62]
SH3BP-5—SH3-domain binding protein 5	Rat2	SINV	[46]
SH3KBP1/CIN85—SH3 domain-containing kinase-binding protein 1	In vitro	SFV, SINV, CHIKV	[62]
	NIH 3T3	EEEV, VEEV, CHIKV	[66]
SK2—Sphingosine kinase 2	HeLa	CHIKV	[74]
SLC25A13—Solute carrier family 25 member 13	NIH 3T3	EEEV, VEEV	[66]
SLC25A5—Solute carrier family 25 member 5/Adenine nucleotide translocator 2	NIH 3T3	EEEV, VEEV	[66]
	Rat2	SINV	[46]
SNX33—Sorting nexin-33	NIH 3T3	EEEV	[66]
SNX9—Sorting nexin-9	NIH 3T3	EEEV	[66]
TRBP—TAR RNA-binding protein	C_7_10	SINV	[18]
Vimentin	BHK-21	SINV	[60]
WDR48—WD repeat-containing protein 48	BHK-21	SINV	[67]
	NIH 3T3	SINV	[66]
YBX1—Y-box-binding protein 1	BHK-21	SINV	[60]
	BHK-21	SINV	[18]
	NIH 3T3	EEEV	[66]
YBX3—Y-box-binding protein 3	NIH 3T3	CHIKV, SINV	[66]
